# A cross-sectional study on the relationship between hematological data and quantitative morphological indices from kidney biopsies in different glomerular diseases

**DOI:** 10.1186/s12882-018-0846-0

**Published:** 2018-03-14

**Authors:** Michelangelo Nigro, Davide Viggiano, Vincenzo Ragone, Tiziana Trabace, Annamaria di Palma, Michele Rossini, Giovambattista Capasso, Loreto Gesualdo, Giuseppe Gigliotti

**Affiliations:** 1 Eboli Hospital “Maria SS Addolorata” p.zza Scuola Medica Salernitana, UOC of Nephrology and dialysis, 84100 Eboli, Italy; 2 Department Medicine and Health Sciences, University of Molise, Campobasso, Italy; 3 Department Cardiothoracic sciences, Second University Naples, 80138 Naples, Italy; 4 Department of Emergency and Organ Transplantation, Nephrology Unit University of Bari Aldo Moro, Bari, Italy

**Keywords:** eGFR, Kidney biopsy, Uric acid, Fractal dimension, Whole section imaging, Quantitative morphological analysis

## Abstract

**Background:**

The classical approach to the analysis of kidney biopsies is based on semi-quantitative scores of the amount of sclerosis, inflammatory infiltrate, fibrosis and vascular damage. However, advanced renal lesions may be accompanied by a paucity of clinical features and, conversely, important clinical abnormalities may be accompanied by minimal histopathological changes. The objective of this study is to correlate new, semiautomatic, quantitative features of kidney biopsies (e.g. fractal analysis) with clinical and hematological parameters using a cross-sectional design.

**Methods:**

Whole slide images from sixty-seven biopsies of patients diagnosed for diabetic nephropathy, hypertensive nephropathy, focal segmental glomerulosclerosis (FSGS) or IgA nephropathy have been used. The images have been semi-automatically quantified in the ImageJ environment, in order to derive the glomerular density, the tubular density, the number of tubules per glomerulus and the fractal dimension of the tubular lumen in the cortex (an index of complexity of the tubular lumen). For each patient, hemato-chemical data have been retrieved, including the uric acid level and the creatinine-based eGFR.

**Results:**

A linear relationship between eGFR and glomerular density was observed in hypertension and FSGS, but not in diabetic nephropathy. Conversely, the eGFR correlated with the tubular density across different glomerular conditions. Moreover, the tubular density was linearly correlated with uric acid levels in different pathological conditions. The fractal dimension of tubular lumen was correlated with the eGFR but only in hypertensive patients. Finally, blood pressure was not correlated to any of the morphological indices tested.

**Conclusions:**

We propose the use of the fractal dimension as a new morphological descriptor of the nephron integrity.

## Background

The introduction of the kidney biopsy into nephrology by Niels Alwall in 1944, and then by Iversen and Brun in 1951, greatly modified the kidney histopathology classification, which was already started in 1914. Since then, great attention was devoted to the interpretation of kidney biopsies, participating in the identification of nephrology as a subspeciality around 1960.

More than 50 years after its invention, the kidney biopsy remains the cornerstone for the classification of vascular/tubule-interstitial nephropathies, and of glomerulopathies [1], but it is still based on subjective, semiquantitative scores [2]. Unfortunately, since the introduction of the bioptic technique by its inventors [3], no great advancements have been made to overcome these limitations.

The need for such advancements stems from the fracture between histological features and clinical data: indeed, advanced kidney lesions may be accompanied by a paucity of clinical impairment and, conversely, important clinical syndromes (e.g. nephrotic syndrome) may be accompanied by irrelevant histological findings (e.g. minimal change disease).

**Fig. 1:**
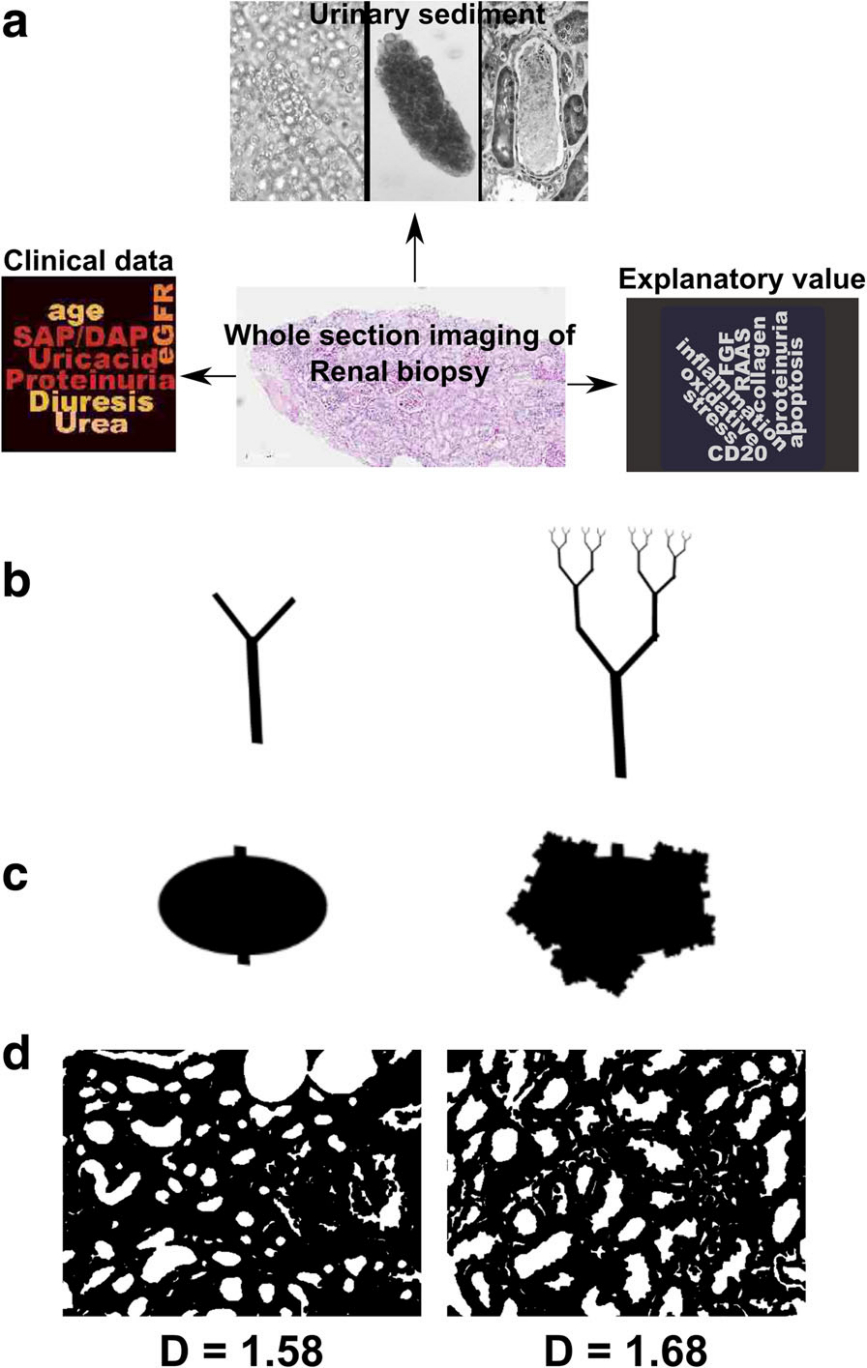
Scheme of image analysis. **a**: desirable correlation between morphological data, clinical data and urinary sediment. **b-d**: fractal analysis. **b**: example of a tree fractal structure, **c**: example of a fractal lumen with recursive undulation of the lumen walls **d**: real images of tubular lumen in two biopsies with different fractal dimensions. **b** and **c** have been drawn using the free internet tool http://recursivedrawing.com/draw.html

This clinical-morphological divergence might underpin a limited ability of clinical/hematological tests to identify renal damage or an, as yet, suboptimal method for the quantification of the renal damage. Ideally, we would need a semi-automatic analysis system of kidney biopsies, with high reproducibility and precision, correlated to the clinical data, the urine sediment data and the ultrasound imaging data; it would be also desirable that these data give information about the prognosis and the therapeutical response (Fig. 1a).

The relatively recent introduction of the Whole Slide Imaging (WSI) technology allows acquiring images of entire sections specimens at the resolution of single cells: this enables us to exploit the unique opportunity to calculate new statistical descriptors of renal sections. Indeed, in this new setting, it is possible to obtain large amount of data concerning the shape and reciprocal position of each morphological object in the section, a so-called high throughput system.

A number of methods have been used to manually extract data from WSI of kidney biopsies [4], while few attempts have been made to automatically analyze kidney sections [5]. An as yet underused approach in renal morphometrics is the application of non-linear techniques to estimate the complexity of the tissue. One possibility to measure the morphological complexity of kidney tissue is the estimation of an index called the fractal dimension (Fig. 1b-d). The fractal analysis has been used in other fields to understand the complex arrangement of natural objects. A fractal is a geometric drawing that is self-similar across different scales. Rivers, trees, beaches, and, specifically, the kidney vascular structure, have fractal appearance because magnifying each of these objects one can see again the same pattern repeated. These structures can be obtained by repeating a simple initial drawing or ‘seed’ (e.g. the simple branching element in fig. 1b left panel) over and over at smaller scales (see fig. 1b, right panel). Similarly, the lumen of a tubule shows a fractal behavior when considering the indented pattern of its profile, which may derive from the repetition of a simple initial pattern (see Fig. 1c). The complexity of the drawing can be expressed with a dimensionless number called fractal dimension, which can be experimentally estimated using specific algorithms (see Fig. 1d).

In the case of the kidney, the fractal dimension of tubules might provide a measure with some taxonomic value [6]. In the present communication we have combined the WSI technology with the use of standard quantitative morphology (e.g. glomerular and tubular density and number of tubules per glomerulus) and non-linear parameters such as the fractal dimension of tubular lumen. Furthermore, we have explored how this association depends on the background pathology of the kidney.

## Materials and methods

This was a retrospective, observational cross-sectional study. Data were taken from the clinical and immunopathological records of the Nephrology Unit in the University of Campania “L.Vanvitelli”; the Nephrology Unit in the Eboli Hospital in the years 2015–2016 and the Nephrology Unit at the Policlinic of Bari “Aldo Moro”.

### Cases

Sixty-seven renal biopsies were analyzed in this pilot study. Inclusion criteria were as follows:

- proteinuria > 1 g/24 h or proteinuria> 100 mg with a creatinine increase of 0.5 mg/dl in the last month with hematuria.
- no use of allopurinol since at least one week (which would confound the meaning of uric acid levels).
- diagnosis of diabetic nephropathy or hypertensive nephropathy or focal segmental glomerulosclerosis or IgA nephropathy. The diagnosis of the subjects derived from histological and immunofluorescence (using standard panels of antibodies) staining of tissue biopsies.
- biopsies containing at least 5 glomeruli. The minimum number of glomeruli to select reliable biopsies was based on the previous experience by Denic et al. [7]. We have further tested whether the selected size of biopsies somehow influenced the quantitative measurements obtained, and found no significant correlation between the area of the section and measured variables (fractal dimension, tubular density, glomerular density and number of tubules per glomerulus).

Moreover, we used the following additional criteria to ensure that all the biopsies were obtained approximately from the same region and orientation in the kidney:

- Biopsies had to contain the capsule. This ensured that the needle did not penetrate too much into the parenchyma, and reduced the effect of depth of the biopsy in the cortex on glomerular density.

Patient parameters (age, sex, weight) and hematological parameters (calibrated creatinine, blood urea nitrogen, and uric acid) have been retrieved for each patient before the bioptic procedure, and the eGFR (estimated by CKD-EPI formula) was calculated. The mean value of the parameters of the patients and the density of glomeruli are reported in Table 1.

**Table 1:** Patients characteristics (classified according to the final diagnosis)

Diagnosis	Hypertensive nephropathy	Diabetic nephropathy	FSGS	IgA	*p* (one way ANOVA)
N	14	18	28	7	
Age (yrs)	46 ± 10	55 ± 13	49 ± 12	35 ± 16	0.007 (IgA < <all other groups)
M/F	9/5	15/3	16/12	4/3	
Body weight (Kg)	71 ± 14	92 ± 19	89 ± 17	65 ± 8	0.004 (IgA < <all other groups)
Urea (mg/dl)	58 ± 21	91 ± 58	46 ± 17	51 ± 24	0.001 (diab> > all other groups)
Uric acid (mg/dl)	6.5 ± 2.2	6.7 ± 2.1	5.7 ± 1.5	6 ± 2	0.45
eGFR (ml/min; CKD-EPI)	49 ± 31	46 ± 31	70 ± 33	82 ± 32	0.019 (diab << all other groups)
Density of glomeruli (n. glomeruli/mm2)	2.6 ± 1.3	2 ± 1	2.3 ± 1.3	2 ± 0.6	0.58

Data represent mean ± SD

### Samples processing and image acquisition

The bioptic specimens have been fixed in Bouin, processed and embedded in paraffin using routine methods. Two and half micron-thick sections (2.5 μ) were stained with hematoxylin-eosin or Periodic Acid Schiff (PAS). The entire sections have been scanned using an Aperio CS2 scanner (Leica): the use of this system of whole slide image (WSI), allows viewing virtually the entire biopsy at high resolution (0.8pixel per micron).

### Computer-assisted image analysis

Section images have been analyzed using the ImageJ free software. Two macros have been developed to semi-automatically perform the image analysis as follows. The images were first converted in 8-bit and smoothed three times to limit the inhomogeneity of the tissue.

The total area of the section was measured in ImageJ after manually setting a threshold to select the section, and then measuring the area. Afterwards, a second threshold was applied to the original image in order to select only the inner side of the tubules.

The thresholding procedure was performed on the basis of the subjective judgment that only the lumen of the tubules was selected, with minimal retention of other areas having low coloration in the tissue (and which were discarded afterwards on the basis of the small size). The manual thresholding was necessary because the intensity of staining among the samples can greatly vary even within the same laboratory. In a pilot study we have also tested the use of automatic local threshold algorithms, which are available in ImageJ environment. However the results were not satisfactory because structures other than the tubular lumen were also obtained. In the future, more specific staining with lower background (such as in immunofluorescence) might be used to fully automatize the process. The thresholding procedure was also retested by a second observer to obtain the reliability index between different observers.

The B/W images were analyzed using the “analyze particles” tool in ImageJ, using a size comprised 200–7000 pixel squares. This corresponded to the total number of tubules in the biopsy section.

The fractal dimension has been measured on the B/W threshold image, with the “Fractal dimension” plugin on a ROI of the biopsy section, thus excluding the border of the section (which could artefactual increase the fractal dimension). We have used the term ‘nephron complexity’ in the manuscript to indicate the fractal dimension of the tubular lumen outlines.

**Fig. 2:**
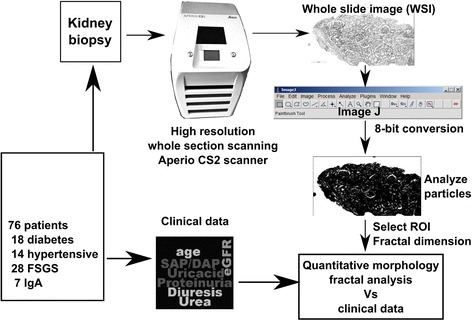
Flowchart of the analysis method of the kidney biopsies

Moreover, the total number of glomeruli per unit area (glomerular density) and number of tubules per glomerulus were also manually counted. A summary of the method is presented in Fig. 2.

The reliability of the measurements has been tested by repeating the analysis by a second observer. The reliability index was then computed on pairs of measurements to find the agreement between observers.

### Histological scoring

Semiquantitative scoring of the sections was conducted according to a previously established protocol [8]. Briefly, sections were blindly evaluated for the following scores: mesangial expansion, interstitial fibrosis and arterial hyalinosis. For each score, five different regions of the biopsy were sampled and evaluated and the mean score used for further analysis. The scores were defined as follow: (i) mesangial expansion: 0 normal, 1 twice normal thickness, 2 three times normal thickness. (ii) interstitial fibrosis: 0 normal, 1 twice normal thickness, 2 three times normal thickness. (iii) arteriolar hyalinosis: 0 normal, 1 incomplete replacement of arteriolar smooth muscle by waxy material, only in few vessels, 1.5 incomplete replacement of most of the arterioles, 2.5 complete replacement of half of the arterioles, 3.5 complete replacement of most of the arterioles.

### Statistical analysis

Statistical analyses were performed using SPSS Statistics for Windows. Glomerular density was calculated from the number of glomeruli (both normal and sclerotic) divided by the area of cortex (mm^2^). To test the presence of differences among the three groups of biopsies (diabetes, FSGS, hypertension, IgA), separate ONE way ANOVAs were executed on each clinical and hematological variable. The outcomes of the study were the correlation between morphological parameters and hematological data. To correlate the clinical parameters to quantitative morphological data, the Pearson’s correlation coefficient was calculated separately for each variable. Significant correlations were then further tested separately in the three groups. The population size was selected in order to find a Pearson’s correlation coefficient above 0.4 between clinical variables and morphological data, with a power of 80%. The rejection level has been set to *p* < 0.05. Possible sources of bias were considered (i) the different size of kidney biopsies and of glomeruli, (ii) the presence of debris inside tubular lumen (which would modify the fractal dimension). To overcome or limit these effects, we used the following cautions, respectively: 1) all counted objects (glomeruli, tubules) have been expressed as number per mm2; moreover, we have tested the effect of section size on the fractal dimension in a pilot experiment 2) we have deliberately excluded sections with the lumen of tubules filled by proteinaceous material which had the same gray intensity of the tubular lumen.

Study size was calculated considering a type I error rate (p) of 0.05, a type II error rate of 0.2 and an expected correlation coefficient of at least 0.35 (which was considered as biologically meaningful), which gave a total sample size of 62 subjects.

## Results

The hematological and anthropometric data of the four study groups are reported in Table 1. The density of glomeruli was not statistically different among groups. The IgA group was significantly younger and with less weight compared to the other groups. Finally, the diabetic subjects showed significantly lower eGFR and greater urea values compared to the other groups. All other clinical values were not significantly different among groups.

The reliability index of the morphological parameters was high for glomerular density (correlation coeff = 0.94, *p* < < 0.001), fractal analysis (corr. Coeff. 0.78, *p* < < 0.001) and tubular density (corr coeff. 0.5, *p* = 0.01). To better understand if the fractal dimension could be more easily represented by more familiar parameters such as the average size of lumens and the density of lumens, we performed a multiple regression analysis using fractal dimension as dependent variable. The results show that tubular lumen size and density explain 26% of the variability of the fractal dimension (R2 = 0.26, *p* = 0.03) with greater explanatory weight given by the average lumen size (standardized partial coefficient beta: 0.52, *p* = 0.01).

The analysis of the linear correlation between morphological parameters (glomerular density, tubular density, number of tubules per glomerulus, fractal dimension of the tubular lumen) and selected hematological values (eGFR, proteinuria, and uric acid, systolic and diastolic pressure) has been carried out pooling all data and is reported in Table 2.

**Table 2:** Pearson’s correlation coefficients and its significance (in parenthesis) among morphological variables and clinical values (bold indicates significant differences)

	Glomerular density	Number of tubules per glomerulus	Tubular density	Fractal dimension of the tubular lumen
Age	− 0.13 (*p* = 0.31)	0.13 (*p* = 0.36)	0.06 (*p* = 0.68)	0.08 (*p* = 0.52)
Body Weight	− 0.11 (*p* = 0.43)	− 0.3 (*p* = 0.06)	0.126 (*p* = 0.41)	−0.12 (*p* = 0.4)
Urea	−0.16 (*p* = 0.21)	− 0.06 (*p* = 0.67)	**− 0.42 (*p*** **= 0.001)**	**−0.33 (*p*** **= 0.007)**
Uric acid	**−0.3 (*p*** **= 0.03)**	0.03 (*p* = 0.84)	**−0.49 (*p*** **< 0.001)**	**−0.25 (*p*** **= 0.05)**
eGFR	**0.27 (*p*** **= 0.03)**	−0.1 (*p* = 0.48)	**0.31 (*p*** **= 0.02)**	**0.27 (*p*** **= 0.03)**
Proteinuria	−0.57 (*p* = 0.67)	−0.097 (*p* = 0.52)	− 0.08 (*p* = 0.55)	0.024 (*p* = 0.85)

As shown in Table 2, both the fractal dimension of tubules and the density of tubules have significant positive correlation with the eGFR and inverse correlation with the level of uric acid and urea. Conversely, the density of glomeruli showed only correlation with the eGFR and uric acid, but not with urea levels.

We further evaluated the eGFR correlation with the density of glomeruli in our samples, by analyzing the correlations separately in the hypertensive, diabetic, FSGS and IgA patients.

**Fig. 3:**
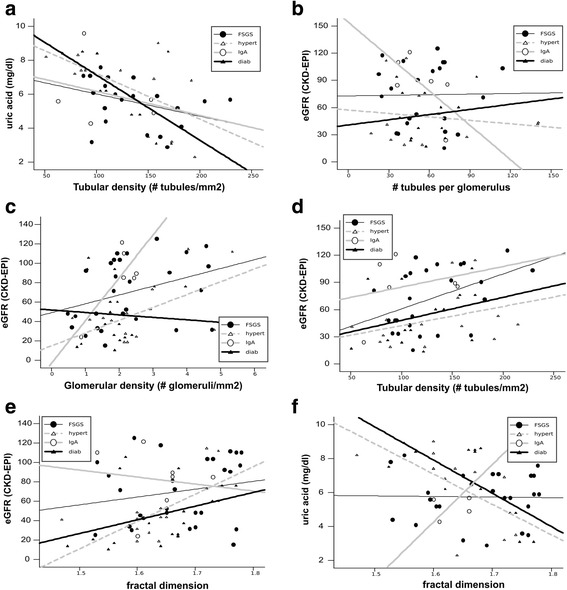
Significant correlations between quantitative morphological data and clinical data in the three groups of patients (FSGS, hypertensive, IgA, diabetes; each dot indicates a subject). The horizontal axis (independent variable) indicates the morphological quantity and the vertical axis the hematological variable. Linear regression lines for each group are superimposed. **a** serum uric acid level as a function of the tubular density. **b**-**e** eGFR as a function of (**b**) the number of tubules per glomerulus, (**c**), the glomerular density, (**d**) the tubular density, (**e**) fractal dimension. **f** serum uric acid as a function of the tubular lumen fractal dimension

The result is reported in Fig. 3 and Table 3, showing a significant positive correlation between eGFR and glomerular density in patients with hypertension (Pearson’s coeff. = 0.58, *p* = 0.03), whereas diabetic patients did not present this correlation. The correlation coefficient between eGFR and glomerular density was very similar in the FSGS group (b = 9 ± 4.8.0, *p* = 0.07), in the hypertensive subgroups (b = 13 ± 5, *p* = 0.03) and in the IgA group (b = 43 ± 18, *p* = 0.07), whereas in the diabetic group was very different (b = − 2.5 ± 7, *p* = 0.71).

**Table 3:** Correlation coefficient in three subgroups between eGFR, uric acid, glomerular density, tubular density and tubular fractal dimension (bold indicates significant differences)

	Hypertensive	Diabetic	FSGS	IgA
eGFR vs glomerular density	**0.58 (*p*** **= 0.03)**	− 0.09 (*p* = 0.7)	0.34 (*p* = 0.07)	0.77 (*p* = 0.07)
eGFR vs tubular density	0.35 (*p* = 0.22)	0.31 (*p* = 0.28)	**0.45 (*p*** **= 0.03)**	0.26 (*p* = 0.61)
eGFR vs fractal dimension	**0.62 (*p*** **= 0.02)**	0.5 (*p* = 0.08)	0.37 (*p* = 0.13)	−0.14 (*p* = 0.77)
Uric acid vs tubular density	**−0.6 (*p*** **= 0.04)**	**−0.63 (*p*** **= 0.05)**	− 0.37 (*p* = 0.09)	0.28 (*p* = 0.65)
Uric acid vs fractal dimension	**−0.6 (*p*** **= 0.04)**	**0.71 (*p*** **= 0.007)**	−0.02 (*p* = 0.93)	0.84 (*p* = 0.07)

The quantitative measurements have been compared with conventional semiquantitative estimates, as shown in Table 4. The results indicate that the tubular density is correlated to the interstitium thickness (corr. Coeff. 0.46, *p* = 0.01), as expected. The glomerular density and the number of tubules per glomerulus are not captured by any of the semiquantitative scores. Finally, the fractal dimension is also correlated to the interstitium thickness, which was also expected given that the fractal dimension of lumen tubules is connected to the alterations in the interstitium.

**Table 4:** Correlation coefficient between quantitative morphological data and semiquantitative histological indices (bold indicates significant differences)

	Glomerular density	Number of tubules per glomerulus	Tubular density	Fractal dimension of the tubular lumen
Mesangial expansion	−0.2 (*p* = 0.3)	−0.22 (*p* = 0.6)	**− 0.5 (*p*** **= 0.004)**	−0.25 (*p* = 0.17)
Interstitium	−0.34 (*p* = 0.06)	0.12 (*p* = 0.54)	**−0.46 (*p*** **= 0.01)**	**−0.57 (** ***p*** **=0.001)**
Vessels	−0.22 (*p* = 0.24)	0.001 (*p* = 0.99)	−0.31 (*p* = 0.08)	−0.15 (*p* = 0.4)

Since the eGFR calculated with the CKD-EPI formula is expected to give larger variability results above 60 ml/min (without bias), we have further tested the correlations found selecting only subjects with a GFR below 60 ml/min. Under these conditions most of the correlations are not any more significant, due to the reduction of the sample size and of the smaller range of eGFR, and, of course, of uric acid and BUN. However, the eGFR is still significantly correlated to the fractal dimension of the sample (corr coeff 0.34, *p* = 0.036, *n* = 38).

## Discussion

The main result of the present paper is that the main morphological variables correlated to eGFR were the tubular density and the fractal dimension of the tubular lumen. Counterintuitively, the eGFR was not correlated to the glomerular density, principally due to the lack of any correlation in diabetic nephropathy.

This result is counterintuitive because the eGFR is supposed to measure the glomerular function and a reduction in the number of glomeruli should result in a smaller eGFR. This might be due to the fact the eGFR is only an estimate and does not reflect true GFR, particularly for filtrates above 60 ml/min [9]. However, the lack of correlation eGFR-glomerular density in diabetic patients deserves some additional consideration. Previous observation on a large datasets (*n* = 1046) showed in healthy subjects (eGFR 81 ml/min) an inverse relation between glomerular density and GFR [10]: subjects with eGFR< 99 had more glomeruli than those with eGFR > 128 ml/min. This has been interpreted as a sign of kidney damage when hyperfiltration is present, which is typical of the first phase of diabetic nephropathy. Accordingly, other reports have shown in diabetic nephropathy a relationship between the structural abnormalities of the glomeruli (rather than their number) with the GFR [11].

We also observed that tubular density predicted for the levels of uric acid in different pathological conditions. This result is in agreement with previous observation that the strongest correlate of renal function is the relative interstitial volume [12]. In the latter work, authors have used an image analysis system to measure the relative interstitial volume, a quantitative proxy of glomerular sclerosis and interstitial fibrosis [12].

Finally, we found that the fractal complexity of tubules (measured by the fractal dimension) had excellent correlation with the eGFR in hypertensive patients. Previous work already showed the application of fractal analysis of kidney sections, limited to normal subjects [13]. Moreover, there has been an attempt to make a semiautomatic measurement of chronic renal damage by recognizing texture modification of tubule-interstitial structures in kidney biopsies [14]. In the latter study, a granulometric function with a circular structuring element granulometry has been on single fields of 35 renal biopsies with different degrees of renal damage. The authors found a positive relationship between granulometric function and glomerular filtration rate was observed (r2 = 0.85).

At variance, we use the entire bioptic section, which ensures that the morphological estimator is not carried out on a non representative field of view, and the use of fractal analysis.

The semi-automatic method proposed in the present communication is objective and susceptible to automatization, in contrast with current scoring systems of kidney biopsies [4]. In an era of precision-based medicine, it is clearly desirable to use semiautomatic, objective measurements, rather than subjective ones.

Our study data have several strengths and limitations that should be noted. First, we have not studied the influence of inflammation or sclerosis on clinical parameters, which have been already addressed elsewhere. Second, this is an observational study and it is not possible to make causal relationship between correlated variables. The new technique here proposed could be used for the study of paraffin-embedded sections stained with classical histological methods, of the major types of kidney diseases (FSGS, diabetic, hypertensive, IgA), and can be easily extended to other sets, although the latter have not been tested here.

It should be noted that sampling errors (different regions of the specimen, different levels of the same core) can always occur when analyzing a small fraction of the kidney, which is of course a general issue of the pathological categorization of the tissue. This problem is partially overcome by the unbiased sampling across different subjects, so that sampling errors should be reflected into higher variability/background noise (and therefore in a smaller statistical significance) and not in modifications of the correlations. The reproducibility of some of the correlations across different pathological classes somehow guarantees that these are unlikely to derive simply from a bias in the sampling. Moreover, in a pilot study based on a smaller set of patients (39 patients) we found similar correlations which, again, confirm their stability. Finally, we did not find a relationship between the parameters and the size of the section, which support the notion of an unbiased sampling of the tissues and the stability of the parameters under study independently from the amount of tissue sampled, when a criteria of a minimum of 5 glomeruli per section and the presence of the capsule are adopted.

One possible caveat in the proposed analysis is the presence of debris within the tubular lumen. We have not directly tested this issue in the samples, and therefore it remains possible that the fractal dimension somehow reflects also the amount of debris in the samples. However, (i) the absence of correlation between the tested variables and the proteinuria (which impacts the amount of intratubular debris) and (ii) the fact that the particles detection algorithm automatically fills holes within the tubular lumen (thereby erasing areas of intraluminal debris) makes unlikely that the fractal dimension is strongly dependent on intratubular debris.

Clearly, future studies should take into account a larger number of clinical parameters and morphological estimators.

## Conclusions

Our results suggest that the modification of eGFR is related to the tubular density. Moreover, we propose the use of the fractal dimension as a new estimator of nephron integrity. Finally, we also suggest that uric acid might bear a better predictive value, compared to eGFR, to identify nephron integrity.

## Abbreviations

eGFR: estimated Glomerular filtration rate
FSGS: Focal Segmental Glomerulo-Sclerosis
WSI: whole slide imaging
